# Age-Related Variation of Bacterial and Fungal Communities in Different Body Habitats across the Young, Elderly, and Centenarians in Sardinia

**DOI:** 10.1128/mSphere.00558-19

**Published:** 2020-02-26

**Authors:** Lu Wu, Tiansheng Zeng, Massimo Deligios, Luciano Milanesi, Morgan G. I. Langille, Angelo Zinellu, Salvatore Rubino, Ciriaco Carru, David J. Kelvin

**Affiliations:** a Division of Immunology International Institute of Infection and Immunity, Shantou University Medical College, Shantou, Guangdong, China; b Department of Biomedical Sciences, University of Sassari, Sassari, Italy; c Institute of Biomedical Technologies, National Research Council of Italy, Segrate, Italy; d Department of Microbiology and Immunology, Dalhousie University, Halifax, Nova Scotia, Canada; University of California, Davis

**Keywords:** 16S rRNA sequencing, ITS1 sequencing, Sardinian, aging, gut microbiota, human microbiome, oral microbiota, skin microbiota

## Abstract

Human body microbes interact with the host, forming microbial communities that are in continual flux during the aging process. Previous studies have mostly focused on surveying a single body habitat to determine the age-related variation in the bacterial and fungal communities. A more comprehensive understanding of the variation in the human microbiota and mycobiota across multiple body habitats related to aging is still unclear. To obtain an integrated view of the spatial distribution of microbes in a specific Mediterranean population across a wide age range, we surveyed the bacterial and fungal communities in the skin, oral cavity, and gut in the young, elderly, and centenarians in Sardinia using 16S rRNA gene and internal transcribed spacer 1 (ITS1) sequencing. We found that the distribution and correlation of bacterial and fungal communities in Sardinians were largely determined by body site. In each age group, the bacterial and fungal communities found in the skin were significantly different in structure. In the oral cavity, age had a marginal impact on the structures of the bacterial and fungal communities. Furthermore, the gut bacterial communities in centenarians clustered separately from those of the young and elderly, while the fungal communities in the gut habitat could not be separated by host age.

**IMPORTANCE** Site-specific microbial communities are recognized as important factors in host health and disease. To better understand how the human microbiota potentially affects and is affected by its host during the aging process, the fundamental issue to address is the distribution of microbiota related to age. Here, we show an integrated view of the spatial distribution of microbes in a specific Mediterranean population (Sardinians) across a wide age range. Our study indicates that age plays a critical role in shaping the human microbiota in a habitat-dependent manner. The dynamic age-related microbiota changes we observed across multiple body sites may provide possibilities for modulating microbe communities to maintain or improve health during aging.

## Abstract (precis)

Site-specific microbial communities are recognized as important factors in host health and disease. To better understand how the human microbiota potentially affects and is affected by its host during the aging process, the fundamental issue to address is the distribution of microbiota related to age. Here, we show an integrated view of the spatial distribution of microbes in a specific Mediterranean population (Sardinians) across a wide age range. Our study indicates that age plays a critical role in shaping the human microbiota in a habitat-dependent manner. The dynamic age-related microbiota changes we observed across multiple body sites may provide possibilities for modulating microbe communities to maintain or improve health during aging.

## INTRODUCTION

The human body and microbes that inhabit it organize together into a supermetaorganism. The distribution of microbes in the human body is largely and primarily determined by body habitats with different ecological niches (1, 2); moreover, it is also influenced by age, lifestyle, ethnicity, and geography (3–6). The resident microbes include not only bacteria but also other microorganisms, such as fungi and archaea. Dysfunction of any members of this community may affect the health of the human body and may cause pathogenic diseases (7, 8). Moreover, the human microbiotas distributed across the body are not isolated from one another but are interconnected as a unit (9). Thus, multikingdom microbiota communities across the human body should not be considered in isolation but as part of an individual pool of interacting communities.

Aging is associated with a wide array of physiological, metabolic, and immunological function declines (10). As a complex and dynamic ecosystem, the human microbiota is subject to continual change in life. Site-specific physiological changes take place during the aging process and are associated with microbial communities also undergoing site-specific changes. For example, the aging-related decrease in sebum in the skin is associated with changes in the skin microbiota (11, 12). Physical and cognitive age-associated changes of a host lead to lifestyle alterations, such as dietary preference changes caused by reducing dental status and decreasing chewing ability (13). Dietary fiber deprivation can change the gut microbiota composition and promote intestinal barrier dysfunction (14). Interestingly, many aging-related clinical issues are closely correlated with the changed microbiota. For example, ulcers in the skin which commonly happen in the physically limited or bedridden elderly were associated with altered skin microbiota (15). A proinflammatory gut microbiota may contribute to the development of atherosclerosis and stroke (16, 17). Moreover, the microbiota also can modulate the aging process (18). The intestinal barrier dysfunction and systemic inflammation promoted by age-related microbial dysbiosis have been observed in mice (19). Therefore, investigating the age-related microbiota change in humans is critical to understanding the interplay between the human microbiota and host during aging.

Aging-related alterations in the gut bacterial community in different cohorts have been demonstrated in previous studies (20–28). The variation may be caused by diverse genetic, dietary, and environmental factors (29–32). Age-related skin bacterial community changes in a Japanese population and a Chinese population have been reported (33, 34). The oral microbiota is emerging as a promising indicator for heath (35, 36). However, the age-related variation in the oral microbiome at the population level has not been fully demonstrated (36, 37). Compared with extensive studies on site-specific bacterial communities in the human body, how aging affects the site-specific fungal communities is poorly understood.

In order to examine the variation of microbiota in different body habitats across different age groups, we recruited 65 subjects from Sardinia, Italy. This cohort formed part of the AKEntAnnos (AKEA) cohort study (38). The composition of the cohort was a healthy young group (age 19 to 33 years), a healthy elderly group (age 68 to 88 years), and a centenarian group (>100 years old). 16S rRNA gene and internal transcribed spacer 1 (ITS1) gene amplicon sequencing methods were carried out to survey the skin, oral, and gut microbiota communities at each site. Our study provides new insights into how age influences the diversity of bacterial and fungal communities in various body habitats in the Sardinian population.

## RESULTS

### 16S rRNA gene and ITS1 gene amplicon sequencing of the microbiota from different body habitats.

To compare community structures and the relationship between bacteria and fungi among body habitats in different age groups, a total of 65 Sardinians from three age groups were recruited (Table 1). For each subject, skin (four different sites, the left palm, right palm, forehead, and umbilicus), oral, and fecal samples were collected. The workflow is shown in Fig. S1 in the supplemental material. A total of 379 16S rRNA and 377 ITS1 quality control amplicon libraries were constructed and sequenced on an Illumina MiSeq platform. More than 9.6 million reads were generated for 16S rRNA sequencing, and 11.2 million reads were generated for ITS1 sequencing. On average, 25,341 clean reads for 16S rRNA sequencing and 29,813 clean reads for ITS1 sequencing were sequenced for each sample. Across all six body sites, we detected members of 30 bacterial phyla assigned to 562 bacterial genera and 6 fungal phyla assigned to 691 fungal genera. The clinical characteristics and health measurements for the three age groups and the total distribution of the bacterial and fungal community taxonomy assignments for each sample are displayed in Data Set S1.

**TABLE 1:** Clinical characteristics of the Sardinian cohort

Characteristic	Data for population (*n*):		
	Centenarians (22)	Elderly (24)	Young (19)
Age (mean ± SD [range]) (yr)	102.0 ± 1.5 (99–107)	77.2 ± 5.9 (68–88)	24.9 ± 3.5 (19–33)
No. of males	6	10	7
No. of females	16	14	12

10.1128/mSphere.00558-19.3 DATA SET S1 The clinical characteristics and health measurements in the three age groups and the distribution of the bacterial and fungal community taxonomy assignments for each sample at the phylum, family, and genus levels. The distribution was calculated based on the rarefied sequences. Taxa with an average number of reads per sample over 1 are shown. Download Data Set S1, XLSX file, 0.5 MB. Copyright © 2020 Wu et al. 2020 Wu et al. This content is distributed under the terms of the Creative Commons Attribution 4.0 International license.

10.1128/mSphere.00558-19.4 FIG S1 Design and workflow of the study. Download FIG S1, EPS file, 0.7 MB. Copyright © 2020 Wu et al. 2020 Wu et al. This content is distributed under the terms of the Creative Commons Attribution 4.0 International license.

### Bacterial and fungal community α diversity in three age groups across the different body sites.

At a normalized sequence depth, the bacterial and fungal community diversity and richness evaluated by the Shannon diversity index and Chao1-estimated operational taxonomic units (OTUs) varied dramatically in different body sites (Fig. S2). Overall, both analyses revealed that the bacteria had a significantly higher α diversity than that of fungi in the same body sites (Fig. S2a; Kruskal-Wallis test, *P* < 0.001). The left and right palms showed similar significantly higher bacterial and fungal community α diversity than did the forehead (Fig. S2b). The α diversity of the fungal communities in the oral cavity and gut was significantly lower than that in the palms (Fig. S2b). The Shannon diversity indexes of the bacterial communities were not significantly different in the gut and oral cavity, and neither were the α-diversity values of the fungal communities (Fig. S2b), while significantly higher Chao1 indexes of the bacterial communities and significantly lower Chao1 indexes of the fungal communities in the gut were observed than what was found with the oral cavity (Fig. S2b).

10.1128/mSphere.00558-19.5 FIG S2 Bacterial and fungal community α diversity in different body sites. (a) Bacterial and fungal community α-diversity indexes evaluated by median Shannon diversity indexes and Chao1 indexes of fungal and bacterial OTUs in 6 different body sites. Error bars represent the median absolute deviation. (b) The comparison of the bacterial and fungal community α-diversity values among 6 different body sites. Statistical significance is indicated as follows: ***, *P* < 0.001; **, *P* < 0.01; *, *P* < 0.05 (Kruskal-Wallis test). L, left palm; R, right palm; F, forehead; U, umbilicus; O, oral cavity; G, gut. Download FIG S2, EPS file, 1.1 MB. Copyright © 2020 Wu et al. 2020 Wu et al. This content is distributed under the terms of the Creative Commons Attribution 4.0 International license.

**FIG 1:**
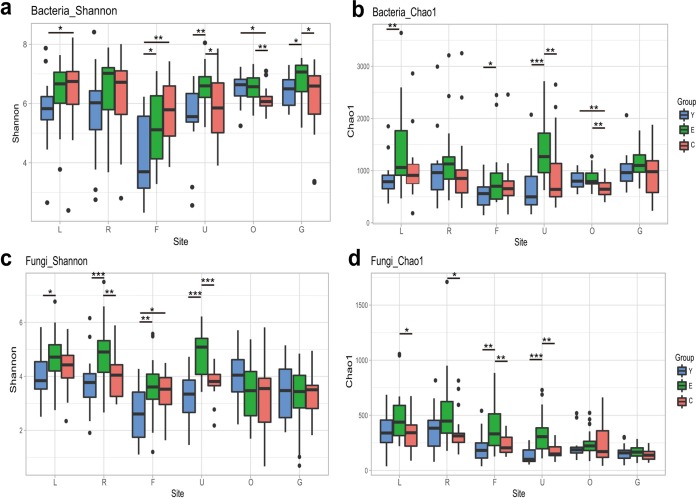
Comparison of bacterial and fungal community α diversity among different age groups for each body site. (a and b) Bacterial community α diversity evaluated by Shannon diversity indexes (a) and Chao1 indexes (b) derived from OTU profiles. (c and d) Fungal community α diversity evaluated by Shannon diversity indexes (c) and Chao1 indexes (d) derived from the OTUs profiles. Box and whisker plots show high, low, and median values, with lower and upper edges of each box denoting first and third quartiles, respectively. Statistical significance is indicated as follows: ***, *P* < 0.001; **, *P* < 0.01; *, *P* < 0.05 (Kruskal-Wallis test). L, left palm; R, right palm; F, forehead; U, umbilicus; O, oral cavity; G, gut.

To further elucidate the age-related α diversity for bacterial and fungal communities, α diversity was compared between the three age groups (Fig. 1a to d). The significance of α diversity between age groups depended on the analyses. For bacterial communities in the gut, no significant difference in Chao1 index values was found among the three age groups, while significantly higher Shannon diversity index values were found in the elderly group than in the young and centenarian groups (Fig. 1a and b). For bacterial communities in the oral cavity, both analyses revealed that the centenarians had significantly lower α diversity than did the young and elderly, who shared similar α diversity (Fig. 1a and b). For bacterial communities in the umbilicus, significantly higher α diversity was found in the elderly than with the young and centenarians (Fig. 1a and b). For the fungal communities in the oral cavity and gut, the variation of α-diversity values between the three age groups was not significant (Fig. 1c and d). For fungal communities in the umbilicus and forehead, significantly higher α diversity was found in the elderly than with the young and centenarians; also, the fungal communities in the palms also displayed higher α diversity in the elderly than did those in the young and centenarians (Fig. 1c and d).

### Bacterial and fungal community β diversity in the different body sites.

**FIG 2:**
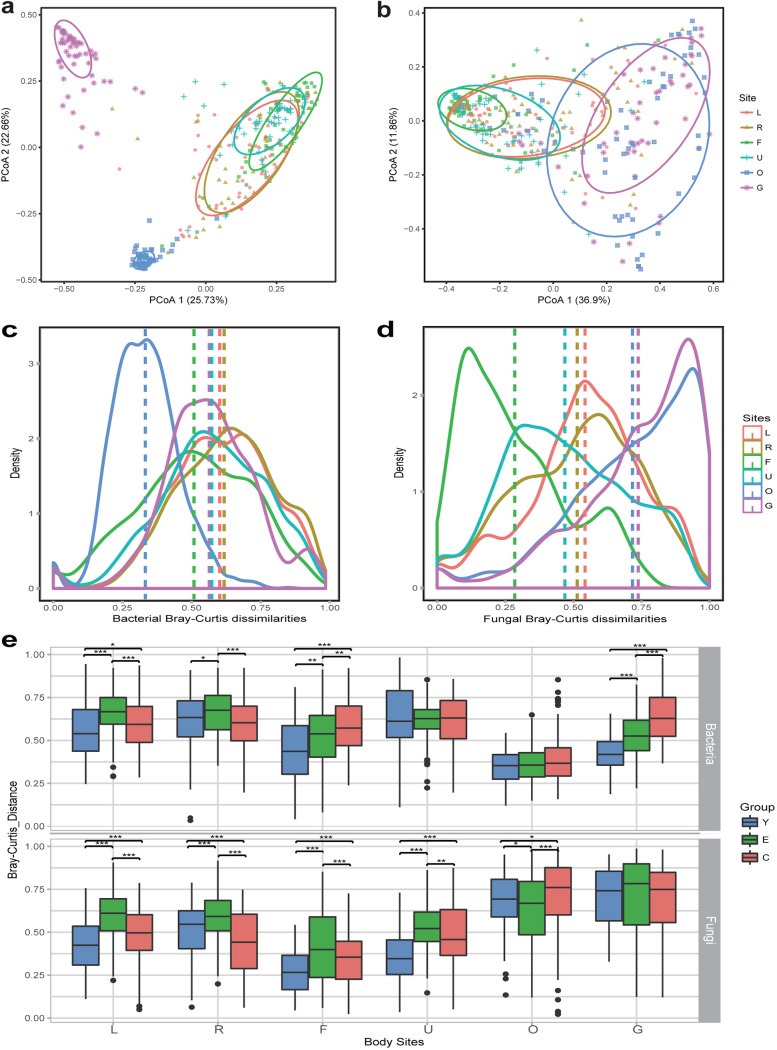
Bacterial and fungal community β diversity in different body sites. (a and b) PCoA of Bray-Curtis distances for the bacterial (a) and fungal (b) communities in each individual across the body sites at the genus level. Samples in each body site are clustered by 95% confidence ellipses. (c and d) Density plots of bacterial (c) and fungal (d) community composition similarities in each body site. The dashed lines correspond to the mean distance of the pairwise comparisons between individuals for each body site. (e) Differences in the similarity of bacterial and fungal community structures among the three age groups in each body site. Community composition similarity was measured based on the Bray-Curtis distance between two samples within each body site at the genus level. Box and whisker plots show high, low, and median values, with lower and upper edges of each box denoting first and third quartiles, respectively. Statistical significance is indicated as follows: ***, *P* < 0.001; **, *P* < 0.01; *, *P* < 0.05 (Kruskal-Wallis test). L, left palm; R, right palm; F, forehead; U, umbilicus; O, oral cavity; G, gut.

Principal-coordinate analysis (PCoA) based on the Bray-Curtis distance of the microbiota compositional profiles at the genus level revealed that the primary clustering of the bacterial community in the body was driven by body site (Fig. 2a). Compared to the skin, where the communities were rather loosely clustered, the gut and oral communities were densely clustered. The Adonis test showed that the bacterial communities were significantly clustered by individual subjects (*R*^2^ = 25%, *P* = 0.001). Meanwhile, the grouping of bacterial communities by body site was also statistically significant (*R*^2^ = 21%, *P* = 0.001), with gut bacterial communities forming a cluster away from bacterial clusters of the skin and oral cavity. The clustering of fungal communities in the PCoA plots was less distinctly separated by body site than that of bacterial communities (Fig. 2b); however, the Adonis test showed that the grouping of fungal communities was statistically significant both for each body site (*R*^2^ = 15%, *P* = 0.001) and in each individual (*R*^2^ = 30%, *P* = 0.001). Overall, although the human microbiota is grouped by body habitats, they are highly specific to an individual.

To assess the effect of different body habitats on community variation, the compositional differences of bacterial and fungal communities among individuals (β diversity) for each body site were assessed (Fig. 2c and d). The distances within the defined body site revealed the different degrees of community composition dissimilarity. The Kruskal-Wallis test revealed that the variation in bacterial community compositions between individuals in the palms was significantly larger than that of the other body sites, while the variation was smallest in the oral cavity (*P* < 0.05). The fungal community compositional variations within the oral cavity and gut were significantly larger than with the skin (Kruskal-Wallis test, *P* < 0.05).

### Bacterial and fungal community β diversity in the different age groups across body sites.

Analysis of similarity (ANOSIM) and a multiresponse permutation procedure (MRPP) test were applied to test statistically whether the microbiota communities were significantly different among the three age groups (Table 2). The tests revealed that the three age groups showed different degrees of subclustering in certain habitats. The skin and gut bacterial communities were statistically different for different age groups, but oral bacterial communities were not statistically different for different age groups. No significant difference among the three age groups was detected for the fungal communities in the oral cavity and gut (ANOSIM test, *R* < 0.05 and *P* > 0.05). The fungal communities for the skin habitats showed a significant difference among age groups, but even so, the group dissimilarities were not sharp (0 < *R* < 0.1). To avoid biases related to the unequal distribution of sexes, permutational multivariate analysis of variance (PERMANOVA), ANOSIM, and MRPP test based on the Bray-Curtis dissimilarity at the genus level were used to look at the sex-related difference in microbiota distributions in each body site (Table S1a). Sex displayed a weak contribution to the variability in microbial distribution.

**TABLE 2:** Statistical evaluation of the differences in bacterial and fungal communities among age groups and body sites

Community (sequencing)	Body site^*a*^	Results for^*b*^:							
		ANOSIM		MRPP test					
		Test statistic	*P* value	Observed Δ	Expected Δ	C Δ	E Δ	Y Δ	*P* value
Bacteria (16S rRNA)	L	0.225	**0.001**	0.75	0.79	0.749	0.806	0.681	**0.001**
	R	0.208	**0.001**	0.76	0.79	0.758	0.798	0.721	**0.001**
	F	0.128	**0.001**	0.65	0.67	0.729	0.618	0.577	**0.001**
	U	0.127	**0.003**	0.76	0.78	0.783	0.738	0.766	**0.001**
	O	0.019	0.185	0.54	0.55	0.598	0.528	0.502	**0.008**
	G	0.176	**0.001**	0.76	0.79	0.819	0.764	0.678	**0.001**
Fungi (ITS1)	L	0.091	**0.002**	0.66	0.68	0.635	0.762	0.546	**0.001**
	R	0.083	**0.009**	0.67	0.7	0.612	0.773	0.616	**0.001**
	F	0.088	**0.003**	0.54	0.57	0.563	0.609	0.428	**0.001**
	U	0.08	**0.015**	0.65	0.67	0.648	0.707	0.572	**0.001**
	O	0.029	0.125	0.84	0.84	0.829	0.848	0.801	0.071
	G	0.04	0.087	0.85	0.85	0.850	0.860	0.790	0.054

a L, left palm; R, right palm; F, forehead; U, umbilicus; O, oral cavity; G, gut.

b ANOSIM and MRPP tests based on the Bray-Curtis distance at the genus level were used to statistically evaluate the differences in bacterial and fungal communities among the three age groups in each body site. The number of permutations is 999. *P* values of <0.05 are highlighted in bold.

10.1128/mSphere.00558-19.1 TABLE S1 Significance tests of the overall microbial community structure. (a) ANOSIM, MRPP, and PERMANOVA based on the Bray-Curtis distance at the genus level to statistically evaluate the difference between the sexes in six body sites for the bacterial and fungal communities. (b) ANOSIM based on the Bray-Curtis distance at the genus level to statistically evaluate the difference between each two age groups in six body sites for the bacterial and fungal communities. (c) Significant clinical parameters associated with gut microbiota profiles. *R*^2^ and *P* values for significant covariates were determined by envfit based on the Bray-Curtis dissimilarity distance of gut bacterial and fungal communities. The number of permutations is 999. L, left palm; R, right palm; F, forehead; U, umbilicus; O, oral cavity; G, gut; C, centenarian; E, elderly; Y, young. Download Table S1, DOCX file, 0.1 MB. Copyright © 2020 Wu et al. 2020 Wu et al. This content is distributed under the terms of the Creative Commons Attribution 4.0 International license.

PCoA based on the Bray-Curtis distance of the microbiota compositional profiles at the genus level was used to visualize bacterial and fungal community structures for the different age groups (Fig. S3). We found that the age group clustering matched the ANOSIM and MRPP test results, as shown in Table 2. More specifically, ANOSIM based on the Bray-Curtis distance at the genus level was applied to statistically evaluate the differences between two age groups in six body sites for the bacterial and fungal communities (Table S1b). We observed that for the bacterial communities, the symmetric palms and forehead showed a similar pattern, where the young and centenarians had clearly separated clusters, while the elderly had clusters that overlapped with the young and centenarians (Fig. S3a to c). In the umbilicus sites, the clustering of the bacterial communities for age groups was slightly different, in that elderly and centenarian communities clustered closely but distinct from those of the young (Fig. S3d and Table S1b). For the fungal communities in the skin, the young and centenarians showed close clustering, whereas the elderly clustered separately (Fig. S3g to j and Table S1b). The bacterial and fungal communities in the oral cavity did not display any age group clustering (Fig. S3e and k and Table 2). However, for the gut, we found that the young and elderly had similar clusters for the bacterial communities which were distinct from that of the centenarians (Fig. S3f and Table S1b). The fungal communities in the gut were not separated by age group (Fig. S3l and Table 2).

10.1128/mSphere.00558-19.6 FIG S3 (a to l) PCoA visualizing the dissimilarities of the bacterial (a to f) and fungal (g to l) community structures for each individual in three age groups for each body site based on Bray-Curtis distances of the relative abundance of each genus. Individuals are identified as spots filled with black (centenarian [C]), red (elderly [E]), and green (young [Y]) in a scatter plot. Each age group is clustered by a 95% confidence ellipse. L, left palm; R, right palm; F, forehead; U, umbilicus; O, oral cavity; G, gut. Download FIG S3, EPS file, 1.9 MB. Copyright © 2020 Wu et al. 2020 Wu et al. This content is distributed under the terms of the Creative Commons Attribution 4.0 International license.

Host health parameters may act as covariates and associate with the host gut microbiota composition. We next used envfit analysis to determine the significant factors that can explain the gut microbiota variance (Table S1c). Several covariates were significantly associated with the gut bacterial community, with the functional independence measure (FIM) and the Mini-Mental State Examination (MMSE) indexes explaining the greatest amount of variance. Conversely, the factors we measured had no significant association with gut fungal community.

To investigate the age-related effects on community variation, the compositional differences of bacterial and fungal communities among individuals (β diversity) in the three age groups were assessed for each body site (Fig. 2e). We observed considerable variation in the bacterial and fungal community structures among individuals in the different age groups, with the exception of the umbilicus and oral cavity bacterial and gut fungal communities (Fig. 2e). For example, the variation in bacterial community compositions between centenarian individuals was significantly larger than those of the elderly and young, and the variation was smallest in the young (Kruskal-Wallis test, *P* < 0.05), while the fungal community compositional variation within each age group was not significantly different in the gut (Kruskal-Wallis test, *P* > 0.05).

### Bacterial and fungal community taxonomic compositions in the three age groups across the different body sites.

**FIG 3:**
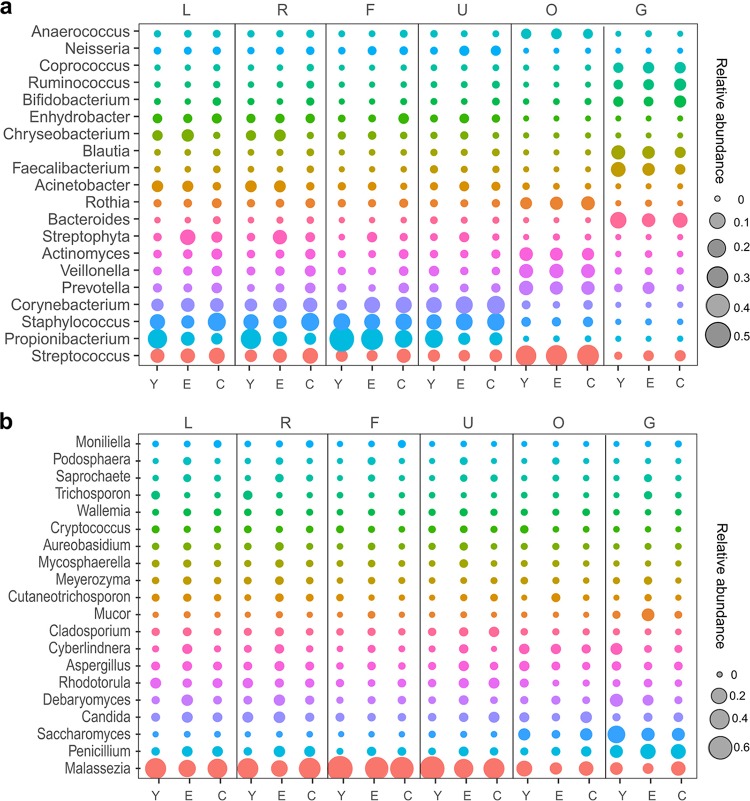
Taxonomic overview of the bacterial and fungal communities in the three age groups across the six body sites. (a and b) The bubble chart shows the top 20 most abundant bacterial genera (a) and fungal genera (b). The sizes of the bubbles refer to the average relative abundance of each genus (listed in the *y* axis) in each of the age groups (listed in the *x* axis). The vertical lines separate different body sites.

To obtain a taxonomic overview of the bacterial and fungal communities, a genus-level relative abundance comparison was performed across the six body sites in the three age groups (Fig. 3 and S4). The resulting profiles showed that each body habitat had a characteristic compositional pattern for both bacteria and fungi (Fig. 3). The dominant bacteria in the skin sites were Propionibacterium, Staphylococcus, and Corynebacterium spp. (Fig. 3a), while the dominant fungi were Malassezia spp. (Fig. 3b). The dominant bacteria in the oral cavity were Streptococcus, Veillonella, Prevotella, Rothia, and Actinomyces spp. (Fig. 3a), while the dominant fungi in the oral cavity were *Malassezia*, Candida, and Saccharomyces spp. (Fig. 3b). The dominant gut bacteria were Bacteroides, Faecalibacterium, Blautia, Coprococcus, and Bifidobacterium spp. (Fig. 3a), whereas Penicillium and *Saccharomyces* spp. were the dominant fungi in the gut (Fig. 3b).

10.1128/mSphere.00558-19.7 FIG S4 Taxonomic compositional features of microbiota among the three age groups in each body site. The bar plot shows the relative abundances of the top 10 most abundant bacterial and fungal genera in each age group. C, centenarian; E, elderly; Y, young. Download FIG S4, EPS file, 2.0 MB. Copyright © 2020 Wu et al. 2020 Wu et al. This content is distributed under the terms of the Creative Commons Attribution 4.0 International license.

Diversification of the bacterial and fungal community compositional signatures in the three different age groups was also observed (Fig. 3 and S4). For example, in the oral cavity, the dominant genus profiles were similar among the three age groups in the bacterial community (Fig. 3a and S4i). The dominant fungal genus profiles in the palms was similar for the young and centenarians but different from that for the elderly, which is in part due to the remarkably low average relative abundance of *Malassezia* spp. in the elderly (Fig. 3b and S4d and f). Several genera also had a wide range of average relative abundance in different age groups. For example, the average relative abundance of *Propionibacterium* spp. in the forehead for the young was 0.54, while it was 0.39 for the elderly and 0.21 for the centenarians (Fig. 3a). *Saccharomyces* spp. had an average relative abundance in the gut for the elderly and centenarians (0.10) similar to that for the young (0.27) (Fig. 3b).

**FIG 4:**
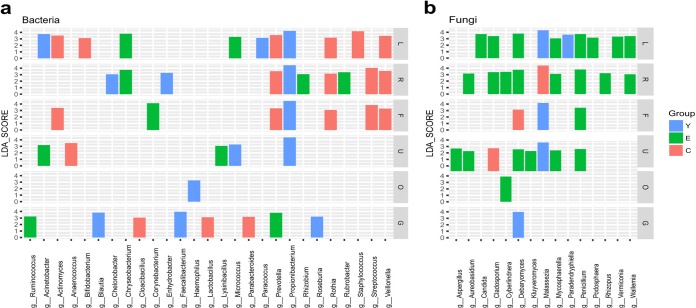
Taxonomic differences were detected using the linear discriminant analysis effect size (LEfSe) algorithm among the three age groups for each body site. (a and b) Only shown are differentially abundant bacterial genera (a) and fungal genera (b) with a linear discriminant analysis (LDA) score of ≥3.0 among the age groups (*P* < 0.05). C, centenarian; E, elderly; Y, young; L, left palm; R, right palm; F, forehead; U, umbilicus; O, oral cavity; G, gut.

To study which bacterial and fungal taxa had significant differences in abundance among the three age groups, the linear discriminant analysis (LDA) effect size (LEfSe) was determined. The results showed that 27 bacterial genera and 15 fungal genera had differential abundance in the three age groups (Fig. 4). For example, overrepresentation of Propionibacterium spp. was observed in the four skin sites (left palm, right palm, forehead, and umbilicus) for the young, while *Prevotella*, *Rothia*, and *Veillonella* spp. were enriched in the left palm, right palm, and forehead for the centenarians. In the gut, *Blautia*, Roseburia, and *Faecalibacterium* spp. were enriched in the young, *Prevotella* and Ruminococcus spp. were enriched in the elderly, and Parabacteroides, Lactobacillus, and Cloacibacillus spp. were enriched in the centenarians. In the oral cavity, Haemophilus spp. were enriched in the young. Interestingly, we observed a significant enrichment of *Bifidobacterium* spp. in the left palms of the centenarians (Fig. 4a). The enrichment of *Bifidobacterium* spp. could be detected in other body sites of centenarians, although not all enrichment reached statistical significance (Fig. S5). Fungi showed accumulation in the skin in the elderly compared with the young and centenarian groups (Fig. 4b). For example, Debaryomyces and *Penicillium* spp. were enriched in palms and umbilicus for the elderly. *Malassezia* spp. were enriched in the left palm, forehead, and umbilicus for the young while in the right palm for the centenarians (Fig. 4b).

10.1128/mSphere.00558-19.8 FIG S5 The boxplots show the relative abundances of *Bifidobacterium* spp. in the three different age groups in each body site for the Sardinians. The significance of variations between age groups was detected by a Kruskal-Wallis test, followed by Dunn’s multiple-comparison test, as follows: **, *P* < 0.01; *, *P* < 0.05. Download FIG S5, EPS file, 1.3 MB. Copyright © 2020 Wu et al. 2020 Wu et al. This content is distributed under the terms of the Creative Commons Attribution 4.0 International license.

### Bacterial and fungal correlations at the community and genus levels.

**FIG 5:**
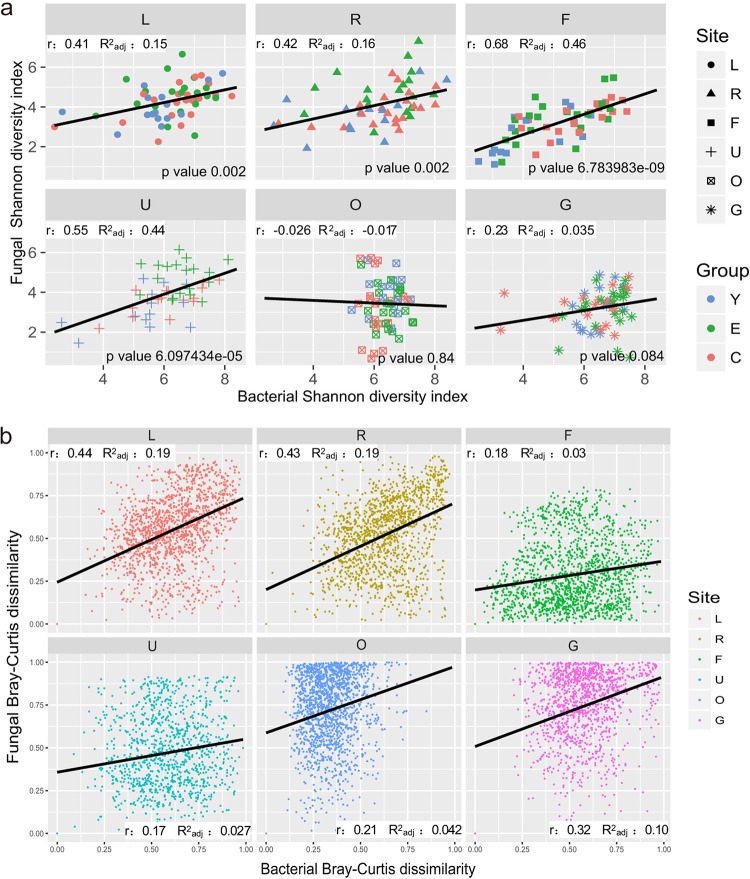
Correlations between bacterial and fungal communities. (a) Correlation of bacterial and fungal community Shannon diversity indexes in each body site. The samples are differently shaped for each body site and colored by age groups labeled by C (centenarian), E (elderly), and Y (young). (b) Correlation of fungal and bacterial Bray-Curtis dissimilarity pairwise comparisons at the genus level within each body site. The adjusted (adj) *R*^2^ of the linear regression analysis and Pearson correlation coefficient (r) are provided for each plot. All *P* values in panel b are <0.0001. L, left palm; R, right palm; F, forehead; U, umbilicus; O, oral cavity; G, gut.

To explore potential correlations between bacterial and fungal community diversity in the human body, community α diversity was evaluated by the Shannon diversity index for each clinical sample within each body site (Fig. 5a). The Shannon diversity values for the bacterial and fungal communities in the gut and oral cavity were not statistically correlated, but a significant correlation was observed in the skin. The linear regression analysis indicated that for the skin, individuals with high diversity in the bacterial community were also associated with a high α diversity in the fungal community. In the gut and oral sites, the α diversity of bacterial and fungal communities appeared to be independent.

To determine the correlations between bacterial and fungal community dissimilarities between any two clinical samples within each body site, linear regression analysis and Mantel tests were performed between fungal and bacterial Bray-Curtis dissimilarity pairwise comparisons (Fig. 5b and Table S2). Interestingly, the greatest positive correlation was detected in the palms, indicating that two individuals with similar bacterial communities were associated with a similar fungal community in palms. For other body sites, the cross-domain community correlations were weak or insignificant.

10.1128/mSphere.00558-19.2 TABLE S2 Correlation of fungal and bacterial Bray-Curtis dissimilarity pairwise comparisons at the genus level within each body site were investigated by a Mantel test. L, left palm; R, right palm; F, forehead; U, umbilicus; O, oral cavity; G, gut. Download Table S2, DOCX file, 0.1 MB. Copyright © 2020 Wu et al. 2020 Wu et al. This content is distributed under the terms of the Creative Commons Attribution 4.0 International license.

Cooccurrence analysis of each body habitat, based on Pearson’s correlation of fungal and bacterial taxonomic relative abundances, provided a preliminary evaluation of major fungus-bacterium genus associations (Fig. S6). It revealed that for different niches, the correlations of the bacterial and fungal taxa were diverse. In the skin, bacteria and fungi exhibit greater numbers of significant bacterium-bacterium, fungus-fungus, and cross-domain correlations, while in the oral cavity, most of those correlations belong to bacterium-bacterium correlations. For the gut, a small number of significant correlations were observed, but the number of cross-domain correlations was greater than those observed in the oral cavity. We also observed that the correlation between the same taxa in different niches can be distinct. For instance, *Candida* and *Malassezia* spp. had a significant negative correlation in the skin and oral cavity, but they did not show a significant correlation in the gut. In the gut, the strongest positive correlation occurred between Meyerozyma and Rhodotorula spp., Ruminococcus and Wallemia spp., and Dialister and Trichosporon spp. In the oral cavity, *Rhodotorula* spp. showed a significant positive correlation with Cryptococcus spp.; however, no significant correlation was observed with *Meyerozyma* spp., which was the case in the gut. A strong negative correlation was found between *Prevotella* and *Streptococcus* spp. in the oral cavity, but only a weak positive correlation was observed in the skin.

10.1128/mSphere.00558-19.9 FIG S6 Correlation between fungal and bacterial genera in different body habitats. The colors of the squares represent the correlation strength between the relative abundances of the top 15 most abundant genera in each individual. Only significant correlations were plotted (*P* < 0.05). “f_”, fungi; “b_”, bacteria. Download FIG S6, EPS file, 2.5 MB. Copyright © 2020 Wu et al. 2020 Wu et al. This content is distributed under the terms of the Creative Commons Attribution 4.0 International license.

### Comparative analysis of the age-related gut bacterial communities in different populations.

To investigate the age-related features of gut bacterial community across populations, we compared our results with two previous studies from Italy and Japan which all targeted the 16S rRNA V3-V4 region (24, 26) (Fig. S7). In the PCoA plot, the distribution of extreme aging individuals in different populations shifts in the same direction away from the young and elderly and was conserved in the Italian and Japanese cohorts, similar to what we observed here (Fig. S7a). In comparing the Sardinian cohort with the cohort from Emilia-Romagna, Italy, we found that several age-related features were shared between the two populations at the genus level for the gut bacterial community (Fig. S7b). For example, lower abundances of *Faecalibacterium* spp. but higher abundances of *Bifidobacterium* spp. in centenarians were observed compared with the young and elderly in both populations.

10.1128/mSphere.00558-19.10 FIG S7 Age-related gut bacterial community structure differences in different age groups across the Sardinian cohort compared to an Emilia-Romagna cohort and a Japanese cohort. (a) PCoA visualized the dissimilarities of the gut bacterial community structures at the genus level for each individual in different age groups in the Sardinian cohort, Emilia-Romagna cohort, and a Japanese cohort based on Bray-Curtis distances of the relative abundance of each genus. Each age group was labeled as S (semisupercentenarians), C (centenarians), 90 (individuals age 90 to 99 years), E (elderly), and Y (young). (b) The average relative abundances of the top 10 most abundant bacterial genera in the gut for different age groups in Sardinian and Emilia-Romagna cohorts. SC/E/Y denotes the Sardinian centenarians/elderly/young, and BC/E/Y denotes the Emilia Romagna centenarians/elderly/young. Download FIG S7, EPS file, 2.5 MB. Copyright © 2020 Wu et al. 2020 Wu et al. This content is distributed under the terms of the Creative Commons Attribution 4.0 International license.

## DISCUSSION

Our study here systematically demonstrated age-related bacterial and fungal community variations in different body sites within a single population. Concurrent analysis of bacterial and fungal communities revealed that the body habitats and age differentially shape these two microbial communities. Our study provides a framework for future mechanistic investigation of age-related microbiota adaptation and interactions between fungal and bacterial communities in the human body.

We further demonstrated that the distribution of the microbiota in Sardinians was largely determined by body site and individual, consistent with the previous studies (2, 39). We also showed the various age-related bacterial and fungal community diversity measures in each body site. For example, the significantly low α and β diversity of bacterial and fungal communities in the forehead of the young could be driven by the highly similar skin microbiome and mycobiome among the young with the presence of predominant species in the communities, while the remarkable high α and β diversity of bacterial and fungal communities in the palms of the elderly indicated their highly personalized and complex community structures. Interestingly, in our cohorts, the centenarians have α diversity similar to that of the young, while the elderly had a higher Shannon diversity than that of the young and centenarians. A previous study in U.S., U.K., and Colombian cohorts had observed a positive association between α diversity and age but not in Chinese cohorts (40). It was also observed in a larger Irish cohort that the elderly displayed greater interindividual variation (β diversity) than did younger adults for gut microbiota (20). The dynamic age-related α- and β-diversity features across populations indicate that how aging shapes the gut microbiota may remain an open question.

Although we showed different degrees of variation in the taxonomic compositions of bacterial and fungal communities among the different age groups, bacterial and fungal communities in the skin sites all displayed an age-related clustering, consistent with findings from a previous study in Japan but differing from a study in China (33, 34). In the four skin sites we surveyed, bacterial communities between young and centenarians are significantly different, while fungal communities were more similar between the young and centenarians but distinct from those of the elderly. The skin bacterial and fungal community variations in different age groups were related not only to age; it is possible to assume additional factors, such as host physiological capabilities, environmental exposure, and different nutritional requirements for bacteria and fungi may contribute to the divergence.

The bacterial and fungal species with significantly different abundances among the three age groups were also identified in the skin. *Propionibacterium* spp. were enriched in the skin for the young compared with the elderly and centenarians, which could correlate with the decrease in the sebaceous gland activity in skin sites associated with aging (11), consistent with previous studies (33, 41). Several groups have explored the direct interactions between the skin microbiota and the host immune system (42, 43). High abundances of potentially pathogenic *Staphylococcus* and *Streptococcus* spp. were detected in the skin sites of centenarians, which suggests that further attention should be paid to the possible infection of these potentially pathogenic genera in centenarians. *Malassezia* spp., as dominant lipophilic fungi in human body, declined in the elderly compared with the young in our study, different from a previous study in a Toronto population where the elderly had the highest abundance of *Malassezia* spp. (44). Intriguingly, the centenarian group showed a higher abundance of *Malassezia* spp. than did the elderly, which suggests that the sebum in the skin was not the only determinant of *Malassezia* sp. colonization.

Evidence shows that specific microbes in the oral cavity are associated with oral health and disease and are even linked with other systemic diseases (45, 46). We found that both the bacterial and fungal communities in the oral cavity did not display distinct age-related clustering, although age-associated oral bacterial community structural changes were found in a Chinese cohort (37).

Among those microbial communities distributed across the body, the gut microbiota is the most comprehensively studied microbial community and has the strongest association with human disease and health (8). Numerous recent studies indicated that the gut microbiota had a major impact on the host metabolic status and can regulate host life span in animal models (47–50). With aging, the physiological decline of the host gradually causes dietary alterations and decreased immunity health, consequently leading to changes in the gut microbiota (51, 52).

Our data confirmed the results found in the NIH Human Microbiome Project (HMP) cohort which identified the basic fungal and bacterial community characteristics in the gut, such as the fungal diversity being lower than bacterial diversity, the independent relationship between the α diversity of the bacterial and fungal communities, and the dominant fungal genera, *Saccharomyces*, *Malassezia*, and *Candida* (53). In our study here, the Sardinian population showed certain specific features different from those of the HMP cohort. The dominant genera in the gut, for example, *Penicillium*, are higher in mean relative abundance in the gut in the Sardinian cohort (>10%) than in the HMP cohort (<4%). On the other hand, the abundance of *Cladosporium* spp. was lower in the Sardinian cohort. The cause of these population differences in the human microbiota is unknown; however, environmental factors such as geography, ethnicity, diet, and lifestyle may be contributing factors (3–6). It must be pointed out that technical differences in collection and processing, as well as analysis of data, may also contribute to the observed differences.

We observed that the young and elderly had similar bacterial gut community compositions, consistent with previous studies (21, 27), but different from the bacterial gut community composition of most of the centenarians. Similar results were also obtained by our previous metagenomics sequencing (28), as well as the results from an Emilia-Romagna (Italy) cohort and a Japanese cohort (24, 26). The separated clustering of the gut bacterial communities in the centenarians across populations suggested that age shaped the gut bacterial community following the same trajectory with the aging process. For example, the low abundances of *Faecalibacterium* and *Roseburia* spp. (23, 24, 26, 54) in centenarians were detected in our study and other studies (23, 24). *Parabacteroides* spp. were enriched in the centenarian cohort in Sardinia and a Chinese centenarian cohort in Sichuan but decreased in another Chinese centenarian cohort in Guangxi (22, 23). *Bifidobacterium* and *Lactobacillus* spp. are believed to have health-promoting properties as probiotics (55, 56). However, previous studies reported the low abundances of *Bifidobacterium* and *Lactobacillus* spp. in the elderly (57, 58). However, high abundances of *Bifidobacterium* and *Lactobacillus* spp. were detected in our centenarians but not the elderly compared with the young, as well as the centenarians from Guangxi, China, and Emilia-Romagna, Italy (22, 24), suggesting an interesting gut microbiota feature of human longevity. Moreover, we also observed the enrichment of *Bifidobacterium* spp. in other body sites of the centenarians in Sardinia. We believe a larger cohort may be needed to confirm the importance and statistical significance of this enrichment in centenarians.

Compared to bacterial communities, the gut fungal communities in Sardinians displayed low α diversity and great individual variation, consistent with the HMP population from Houston, TX (53). However, diverse dominant species among populations were also observed. For example, *Penicillium* and *Debaryomyces* spp., which are found in fermented foods but cannot grow in the gut niche (59), had a higher prevalence in Sardinians than in the HMP population and may correspond to the high consumption of cheese in Sardinians (60). Besides, *Debaryomyces* spp. were significantly enriched in the young group compared with the elderly and centenarians in Sardinia. The variation in *Debaryomyces* spp. may partly be caused by the dietary preferences for different age groups. Previous studies have shown that diet can modulate the fungal communities in the murine gut (61). The subjects dominated by resident fungi may be associated with a diet enriched of fungi, further suggesting that caution should be made in the microbiome studies because separating the viable microbes from the nonliving microbes and resident from transient microbes can be difficult (62).

Our preliminary survey of the associations between clinical parameters and gut microbiota indicated that host health status-related factors such as FIM and MMSE could explain the greatest amount of variance among the three age groups of bacterial community, consistent with a previous study that also showed the correlation between gut microbiota and health (25). Moreover, the significant association between mini nutritional assessment (MNA) and gut bacterial community suggests that diet and nutrition could also act as important covariates contributing to the variance of the microbiome composition. Diet is considered one of the main determinants of the gut microbiota (63). Further longitudinal studies tracing how aging shapes the gut microbiota with the dietary and environmental factors will be necessary to determine the role those microbes play in the gut during aging.

With an integrated view of microbiota in different body habitats, our preliminary evaluation of the correlation between fungi and bacteria across body sites at both the community and genus levels revealed that the correlations were site and population specific. At the community level, we detected positive correlations between the Shannon diversity of fungal and bacterial communities in each individual in all four skin sites but not in the oral cavity or the gut. This finding is consistent with previous studies of the skin microbiota in a Hong Kong cohort and the HMP gut microbiota study in a Houston, TX, cohort (53, 64). We detected the greatest significant positive cross-domain correlations in community composition dissimilarities in the palms. Interestingly, a skin microbiome study in a Hong Kong cohort also identified significant positive cross-domain correlations in the skin, especially in the palms (64). Although it seems that those cross-domain correlations are shared across populations, the underlying mechanisms are currently unknown. At the genus level, lipophilic *Malassezia* and *Propionibacterium* showed a significant strong positive association in the skin, reflecting their similar nutrition requirements; in the mucosal habitats, however, the correlation was not statistically strong. Furthermore, in the skin, Actinomyces spp. were positively correlated with *Staphylococcus* and *Streptococcus* spp., but in the oral cavity, the correlation was negative. Also, for different populations, the cooccurrence pattern was different. In our study, a strong negative association was detected between *Prevotella* and *Streptococcus* spp. in the oral cavity, consistent with findings from an Italian study (65), while in a Japanese study, the correlation was positive (36). In the gut, the association relationship we observed, such as the positive association between *Faecalibacterium* and *Saccharomyces* spp., was also found in a U.S. study (61), although the correlation we detected based on the cooccurrence did not necessarily mean a true biological interaction between the species. Further cultivation assays are needed to gain insights into the importance and mechanisms of these correlations.

In summary, our study provides a picture of the diverse features of bacterial and fungal communities in different body sites and refines our understanding of the age-related human microbial community variations. However, to better understand the underlying mechanisms of age-related microbiota variations, we should focus more on the detailed functional descriptions of characteristics of diet, living conditions, community populations, as well as individual physiological characteristics and measurement of the variables for microbial ecological niches (e.g., pH range, dissolved oxygen range, temperature range, salinity range, etc.). Fundamental questions will be addressed about the age-related adaptation and diversification of the human microbiota under controlled environments with well-defined niches.

## MATERIALS AND METHODS

### Subject recruitment and clinical information collection.

We recruited 65 subjects in Sardinia, Italy, as a part of the AKEA project, which studies the extreme longevity in Sardinia (38). Ethics approval was provided by the institutional local ethics committee of the Azienda Sanitaria Locale N.1 of Sassari, Italy. The donors were volunteers recruited from the longevity AKEA project, and participants signed a written consent form. Subjects were divided into three age groups, young, elderly, and centenarians. Exclusion criteria for the young group and the elderly group included the following: (i) history of chronic medical conditions (diabetes and hypertension), (ii) the use of antimicrobial medication (antibiotic or antifungal treatments) 1 year before sampling, and (iii) with chronic dermatologic diseases (psoriasis, atopic dermatitis, vitiligo, or urticaria). Clinical and nutritional data were collected as described in the AKEA study (38).

### Sample collection and DNA extraction.

**(i) Skin samples.** To maximize microbial load, no prior cleaning of the skin was needed before sample collection. The skin samples were collected by professional staff with sterilized swabs (Catch-All sample collection swabs) at four different sites, including the forehead, two palms, and the umbilicus area. A 5- by 5-cm^2^ area of skin was gently rubbed 10 times using a swab premoistened with sterilized enzyme lysis buffer. Each swab tip was placed into an Eppendorf tube containing 200 μl enzyme lysis buffer. The samples were kept on ice while shipping to the lab and were stored at −80°C. Negative-control specimens were collected by exposing swabs to room air and then were processed with the samples. Extracted DNA from the tip of the swabs was performed according to the manual’s instructions for the DNeasy blood and tissue extraction kits, with some modification. In brief, the samples in the lysis buffer were incubated at 37°C for 2 h. Five-millimeter zirconia beads (0.4 g; Sigma) were added. Then, each sample was subjected to a bead-beating step using multivortex V-32 (Biosan) at a maximum of 3,000 rpm for 30 min. Twenty-five microliters of proteinase K was added and incubated at 56°C for 30 min. Then, samples were heated at 95°C for 5 min, followed by ice for 1 min, and performed according to the DNeasy blood and tissue extraction kit protocol. The DNA was eluted with 200 μl Tris-EDTA (TE) buffer. The final DNA concentration was determined using an ND-1000 spectrophotometer (NanoDrop Technologies).

**(ii) Oral samples.** Participants were asked to avoid eating or drinking for 1 h prior to oral cavity sampling. Subjects were asked to let saliva collect in the mouth for at least 1 min. The subject was then asked to drool into a sterilized labeled 50-ml collection tube. This process may be repeated multiple times in order to collect larger volumes of saliva (2 to 5 ml). For the centenarians, it was difficult to collect the saliva, so oral washing samples were collected instead. Centenarians were asked to swish vigorously with 10 ml sterilized water for 30 s and then to expectorate into the tube. The wash was repeated twice. After shipping the samples on ice to the lab, they were centrifuged at 7,500 rpm for 10 min, the supernatant was discarded, and the DNA was extracted from the sediment following the same protocol as with skin samples.

**(iii) Stool samples.** Fecal samples were collected by the participants at home. Participants were provided with a sterilized stool specimen collection tube. After passing stool, a spoon was used to collect about a 1-g stool sample by scraping off the outer layer of solid feces and collecting the central part into the tube. Samples were immediately frozen at home at –20°C and collected by laboratory personnel within 6 weeks. Long-term storage of samples was in a –80°C freezer located at the University of Sassari. DNA was extracted from stool samples according to the manual instructions for the QIAamp DNA stool minikit (Qiagen), with some modifications. In brief, 200 mg of stool was suspended in 1.4 ml of buffer ASL, and 0.4 g of 5-mm zirconia beads (Sigma) was added. Then, each sample was subjected to a bead-beating step using Biosan at a maximum of 3,000 rpm for 30 min. Samples were heated at 95°C for 5 min and then centrifuged for 5 min at 13,000 rpm to pellet the stool particles. Next, 1.2 ml of supernatant was collected, the InhibitEX tablet was added, which was followed by incubation at room temperature (RT) for 1 min and centrifugation at 13,000 rpm for 3 min; then, 15 μl of proteinase K and 200 μl of buffer AL were added to 200 μl supernatant and incubated at 70°C for 10 min. Two hundred microliters of absolute ethanol was then added to the mixture, vortexed, and loaded on QIAamp mini spin columns. The columns were washed with buffer AW1 and buffer AW2, as per the QIAamp DNA stool minikit instructions. The DNA was eluted with 200 μl TE buffer. Finally, the DNA concentration was determined by using an ND-1000 spectrophotometer (NanoDrop Technologies).

### Library construction and sequencing.

Procedures for 16S rRNA and ITS1 library generation were performed as previously described (66). Briefly, the V3-V4 regions of the 16S rRNA gene and ITS1 gene were amplified using an improved dual-indexing approach. The primers for 16S rRNA amplification were 5′-ACTCCTACGGGAGGCAGCAG-3′ and 5′-GGACTACHVGGGTWTCTAAT-3′ (67), and for ITS1 amplification, the primers were 5′-GTAAAAGTCGTAACAAGGTTTC-3′ and 5′-GTTCAAAGAYTCGATGATTCAC-3′ (41). 16S rRNA and ITS1 libraries were, respectively, normalized and pooled using the SequalPrep normalization plate kit (Invitrogen) prior to sequencing on an Illumina MiSeq platform.

### Bioinformatics for 16S and ITS1 sequencing.

Raw sequence reads were first trimmed by removal of the barcodes and linker sequences. Then, VSEARCH was used for truncation of the reads not having an average quality of 15 based on the phred algorithm. Reads with less than 75% of their original length were removed (68). Further read processing was performed using QIIME (version 1.9.1) (69). Reads were clustered into operational taxonomic units (OTUs) at 97% similarity using the open-reference strategy by uclust based on the Greengenes database (2013_8 version) for 16S rRNA and UNITE fungal ITS database (version 7.2) (70, 71). The first cluster seed was chosen as the representative sequences for the OTUs. uclust for 16S rRNA sequencing and BLAST for ITS1 sequencing were used for taxonomic assignment of the representative sequences. Alignment of representative sequences was performed using PyNAST for 16S rRNA sequencing and MUSCLE for ITS1 sequencing. The aligned sequences were filtered and phylogenetic trees constructed using FastTree. Following rarefaction, an OTU table was generated in QIIME to minimize the difference in sequencing depths across samples (5,000 reads per sample for 16S rRNA libraries, 10,000 reads per sample for ITS1 libraries, and 10,000 reads per sample for cross-population gut bacterial community comparison). Rarefaction OTU counts were binned into genus-level taxonomic groups according to the taxonomic assignments described earlier. α-diversity indexes, including Chao1 richness and the Shannon diversity index, were calculated using QIIME.

### Statistical analysis.

All statistical analyses were performed using the R software (version 3.4.2). Multivariate analysis of community diversity employing PCoA was performed using vegan and visualized using ggplot2. A Bray-Curtis distance matrix was used as the dissimilarity index. LEfSe was performed to detect differentially abundant taxa between age groups using the default parameters (72). Only those taxa that showed a *P* value of <0.05 and the threshold logarithmic LDA score of ≥3 were ultimately considered. Kruskal-Wallis tests followed by Dunn’s multiple-comparison test were used to determine whether significant differences existed among multiple groups (73). Linear regression was used to test the correlation between two variables (Shannon diversity index and Bray-Curtis distances). Discrimination among groups was detected by MRPP, ANOSIM, and the Adonis method in vegan in R. All permutation tests (i.e., MRPP, ANOSIM, Adonis, and the Mantel test) were conducted using 999 permutations. The significance and explained variance of covariate (referred to as clinical parameters in Table S1c) were determined using the envfit function in vegan.

### Data availability.

The data sets generated and analyzed during the current study are available in the European Nucleotide Archive (ENA) under study accession number PRJEB25916.
