# Supplementary material for: Age-Related Variation of Bacterial and Fungal Communities in Different Body Habitats across the Young, Elderly, and Centenarians in Sardinia
Source: mSphere. 2020 Feb 26;5(1):e00558-19. doi: 10.1128/mSphere.00558-19 (PMC7045387; doi:10.1128/mSphere.00558-19)
Supplement: TABLE S1 [file mSphere.00558-19-st001.docx]

**Table S1a**

| Communities | Sites | ANOSIM test | | MRPP test | | PERMANOVA  (Adonis) test | | |
| --- | --- | --- | --- | --- | --- | --- | --- | --- |
|  |  | statistic **R** | p-value | **A** | p-value | **F** | **R^2^** | p-value |
| Bacteria (16S) | L | -0.03829 | 0.709 | 0.007628 | 0.057 | 1.5928 | 0.02918 | 0.106 |
|  | R | -0.0892 | 0.947 | 0.005942 | 0.109 | 1.3112 | 0.02506 | 0.209 |
|  | F | -0.0375 | 0.813 | 0.00468 | 0.193 | 1.4802 | 0.02621 | 0.139 |
|  | U | -0.07992 | 0.977 | 0.002008 | 0.257 | 0.98457 | 0.01858 | 0.465 |
|  | O | 0.02098 | 0.301 | -4.271e-05 | 0.41 | 0.68379 | 0.01165 | 0.687 |
|  | G | -0.021 | 0.649 | 0.004429 | 0.09 | 1.5067 | 0.02575 | 0.083 |
| Fungi (ITS1) | L | -0.04047 | 0.797 | 0.0114 | **0.038** | 2.3143 | 0.03598 | **0.034** |
|  | R | -0.05736 | 0.908 | -0.007183 | 0.98 | 0.26005 | 0.00424 | 0.985 |
|  | F | -0.09238 | 0.974 | 0.02066 | **0.01** | 3.2575 | 0.05069 | **0.014** |
|  | U | -0.01394 | 0.573 | 0.0156 | **0.025** | 2.5574 | 0.04522 | **0.028** |
|  | O | 0.06149 | 0.093 | 0.001959 | 0.306 | 1.2437 | 0.02173 | 0.27 |
|  | G | 0.009997 | 0.383 | 0.001012 | 0.379 | 0.95686 | 0.01954 | 0.45 |

**Table S1b**

| Communities | Sites | CvsE ANOSIM test | | CvsY ANOSIM test | | EvsY ANOSIM test | |
| --- | --- | --- | --- | --- | --- | --- | --- |
|  |  | statistic R | p-value | statistic R | p-value | statistic R | p-value |
| Bacteria (16S) | L | 0.1965 | **0.001** | 0.3327 | **0.001** | 0.1609 | **0.004** |
|  | R | 0.2138 | **0.001** | 0.3771 | **0.001** | 0.08081 | 0.076 |
|  | F | 0.08329 | **0.022** | 0.2465 | **0.001** | 0.05653 | 0.09 |
|  | U | 0.00594 | 0.362 | 0.1408 | **0.014** | 0.1277 | **0.016** |
|  | O | 0.01194 | 0.247 | 0.06423 | 0.057 | -0.0148 | 0.593 |
|  | G | 0.2101 | **0.001** | 0.2344 | **0.001** | 0.04792 | 0.135 |
| Fungi (ITS1) | L | 0.09939 | **0.012** | 0.01184 | 0.287 | 0.1687 | **0.01** |
|  | R | 0.1576 | **0.003** | -0.0016 | 0.421 | 0.2078 | **0.002** |
|  | F | 0.04532 | **0.04** | 0.01977 | 0.224 | -0.0097 | 0.531 |
|  | U | 0.07865 | 0.055 | 0.03497 | 0.122 | 0.1508 | **0.01** |
|  | O | 0.07525 | **0.041** | -0.0132 | 0.58 | 0.09483 | **0.032** |
|  | G | 0.01003 | 0.356 | 0.07773 | 0.107 | 0.02882 | 0.263 |

**Table S1c**

| Communities | Covariates | EnvFit test | |
| --- | --- | --- | --- |
|  |  | **R^2^** | p-value |
| Gut bacterial community | **Age** | 0.3851 | **0.001** |
|  | BMI^a^ | 0.0743 | 0.133 |
|  | **MNA^b^** | 0. 2605 | **0.002** |
|  | **Drug^c^** | 0.3179 | **0.001** |
|  | **FIM^d^** | 0.5002 | **0.001** |
|  | **MMSE^e^** | 0.5190 | **0.001** |
| Gut fungal community | Age | 0.0137 | 0.700 |
|  | BMI^a^ | 0.0015 | 0.957 |
|  | MNA^b^ | 0.0062 | 0.861 |
|  | Drug^c^ | 0.0089 | 0.780 |
|  | FIM^d^ | 0.0549 | 0.227 |
|  | MMSE^e^ | 0.0215 | 0.563 |

^a^ Body Mass Index, ^b^ The Mini Nutritional Assessment, ^e^ The Mini-Mental State Examination,^c^ Number of drugs taken per day, ^d^ The Functional Independence Measure.
