# Supplementary material for: Age-Related Variation of Bacterial and Fungal Communities in Different Body Habitats across the Young, Elderly, and Centenarians in Sardinia
Source: mSphere. 2020 Feb 26;5(1):e00558-19. doi: 10.1128/mSphere.00558-19 (PMC7045387; doi:10.1128/mSphere.00558-19)
Supplement: TABLE S2 [file mSphere.00558-19-st002.docx]

| Body sites | Mantel r statistic | p-value |
| --- | --- | --- |
| L | 0.3331 | **0.001** |
| R | 0.33803 | **0.001** |
| F | 0.12436 | 0.122 |
| U | 0.03653 | 0.696 |
| O | 0.05528 | 0.356 |
| G | 0.12172 | 0.051 |
